# Tin, The Enabler—Hydrogen Diffusion into Ruthenium

**DOI:** 10.3390/nano9010129

**Published:** 2019-01-21

**Authors:** Chidozie Onwudinanti, Ionuţ Tranca, Thomas Morgan, Shuxia Tao

**Affiliations:** 1 Center for Computational Energy Research, DIFFER—Dutch Institute for Fundamental Energy Research, 5612 AJ Eindhoven, The Netherlands; c.k.onwudinanti@differ.nl; 2 Department of Mechanical Engineering, Eindhoven University of Technology, 5600 MB Eindhoven, The Netherlands; i.tranca@tue.nl; 3 Plasma Material Interactions, DIFFER—Dutch Institute for Fundamental Energy Research, 5612 AJ Eindhoven, The Netherlands; t.w.morgan@differ.nl; 4 Center for Computational Energy Research, Department of Applied Physics, Eindhoven University of Technology, 5600 MB Eindhoven, The Netherlands

**Keywords:** DFT, surface, hydrogen, ruthenium, tin, transition state, electronegativity, bond order

## Abstract

Hydrogen interaction with ruthenium is of particular importance for the ruthenium-capped multilayer reflectors used in extreme ultraviolet (EUV) lithography. Hydrogen causes blistering, which leads to a loss of reflectivity. This problem is aggravated by tin. This study aims to uncover the mechanism via which tin affects the hydrogen uptake, with a view to mitigation. We report here the results of a study of hydrogen interaction with the ruthenium surface in the presence of tin using Density Functional Theory and charge density analyses. Our calculations show a significant drop in the energy barrier to hydrogen penetration when a tin atom or a tin hydride molecule (SnH_x_) is adsorbed on the ruthenium surface; the barrier has been found to drop in all tested cases with tin, from 1.06 eV to as low as 0.28 eV in the case of stannane (SnH_4_). Analyses show that, due to charge transfer from the less electronegative tin to hydrogen and ruthenium, charge accumulates around the diffusing hydrogen atom and near the ruthenium surface atoms. The reduced atomic volume of hydrogen, together with the effect of electron–electron repulsion from the ruthenium surface charge, facilitates subsurface penetration. Understanding the nature of tin’s influence on hydrogen penetration will guide efforts to mitigate blistering damage of EUV optics. It also holds great interest for applications where hydrogen penetration is desirable, such as hydrogen storage.

## 1. Introduction

Hydrogen interacts with metal surfaces in many varied and important technological applications. This occurs in catalysis [1,2], as well as hydrogen separation [3,4], storage [5], and sensors [6]. The ability of the small atom to permeate metals and change their properties gives hydrogen–metal systems added significance; for instance, embrittlement of metals remains an obstacle to the transport and storage of hydrogen [7,8]. In this case, as in separation and purification, diffusion into the subsurface and bulk of the metal is quite important [9]. This interaction has been studied extensively, particularly with transition metals, and the field maintains a high level of scientific interest and relevance [10,11,12,13]. The addition of a second metal creates a so-called bimetallic surface. This often introduces significant changes to the electronic structure and characteristics of that surface relative to the single metal [14,15,16,17], with consequences for its technological applications.

The use of ruthenium on reflective optics is one such application, with a bimetallic system interacting with hydrogen. The multi-layer reflectors used in the optical systems of extreme ultraviolet (EUV) lithography employ a ruthenium capping layer [18,19]; this surface may be contaminated by tin debris from the laser-pulsed tin plasma, the source of the EUV photons. Hydrogen used for cleaning the optics comes into contact with the surface and debris. It may penetrate the surface and diffuse to the interfaces of the multi-layer structure, where it recombines to form pockets of gas. When these pockets reach a critical pressure, the layers separate, which results in blistering of the mirror and loss of reflectivity [20,21]. This process appears to be facilitated by tin [22].

Ruthenium has been the subject of many studies [23], as it is used in diverse chemical processes. The Ru-Sn bimetallic catalyst has been studied as a promising option for selective hydrogenation of the carbonyl group [24,25], which is important in the production of unsaturated alcohols [26]. It is also a candidate for hydrogen production via glycerol steam reforming [27]. Hydrogen does not readily permeate ruthenium [28], but the existence of subsurface hydrogen has been demonstrated in ruthenium [29,30] and other metals [31], and it may be considered a precursor to dissolved or hydridic hydrogen in the bulk.

On the one hand, the undesirable effects of hydrogen penetration, i.e., blistering of the reflector, make it necessary to study the penetration process and possible solutions. On the other, the facilitation of diffusion into the ruthenium crystal opens the door to potential applications such as separation and purification. The latter is made especially attractive by the significantly lower cost of ruthenium and tin relative to palladium.

In this study, we focus on the tin-mediated hydrogen penetration of the Ru(0001) surface. Using Density Functional Theory (DFT), we demonstrate that tin affects the energy barriers to diffusion in a manner that facilitates penetration into the ruthenium. We show that adsorbed tin atoms and tin hydrides cause hydrogen to bond to the surface with a more compactly distributed charge, and also cause charge accumulation on the metal surface. The result is a lower energy barrier to subsurface diffusion.

The article is organised as follows: in the next section, we present details of the computational methodology employed. Thereafter, we report the results of calculations for hydrogen on the Ru(0001) surface, and in the ruthenium subsurface and bulk. A number of relevant ruthenium-tin-hydrogen interactions are then considered, and we discuss the changes observed upon introducing tin. Finally, we present a comprehensive analysis of the electronic charge density distributions in the examined interactions, and discuss the implications of the results.

## 2. Computational Methods

### 2.1. DFT Calculations

The results presented in this work are based on computations performed within the framework of DFT, as implemented in the Vienna Ab Initio Simulation Package (VASP) [32,33,34]. The calculations were performed with the generalized gradient approach as proposed by Perdew, Burke, and Ernzerhof (PBE) [35], with the following key convergence parameters: a kinetic energy cutoff of 400 eV, residual force criterion of 1 × 10 ${}^{-2}$ eV/Å, and a 1 × 10 ${}^{-5}$ eV energy convergence criterion. Slab calculations were performed with a (9×9×1)Γ-centred *k*-points grid, while bulk calculations were done with a (9×9×9) grid; all atoms were allowed to relax in the optimization process. In order to account for long-range dispersive interactions, all calculations were performed with the DFT-D3 dispersion correction proposed by Grimme et al. [36]. Transition state calculations were carried out using the Climbing Image Nudged Elastic Band (CINEB) algorithm [37], with a force criterion of 1 × 10 ${}^{-2}$ eV/Å and three (3) intermediate geometries for the transition state search.

The calculated lattice parameters for hexagonal close-packed (hcp) ruthenium are a=2.69 Å and c/a=1.58, which are in good agreement with experimental results, 2.71 Å and 1.58, respectively [38]. The surface is modelled by a slab of seven layers using a (2×2) cell, with ∼15 Å of vacuum between the periodic images in the *z*-direction. The number of layers and the vacuum height were found to give accurate results at reasonable computational cost—the calculated surface energy for the (2×2) cell changes by less than 2% from 5 layers to 11 layers.

Lattice parameters a=5.82 Å and b=3.17 Å for solid tin in the β-Sn crystal structure were found to be in good agreement with measured values, 5.83 Å and 3.18 Å, respectively [38]. Slab calculations for Sn(001) and Sn(010) surfaces were performed with (1×1×3) and (1×3×1) cells of 7-layer slabs, with ∼15 Å vacuum.

For hydrogen, the energy of adsorption is computed per the definition

(1) $$E_{ads}=\frac{1}{n}\left(E_{nH,surf}-E_{surf}-\frac{n}{2}E_{H_{2}}\right),$$

where $E_{nH,surf}$, $E_{surf}$, and $E_{H_{2}}$ stand respectively for the total energies of the ruthenium slab with *n* adsorbed hydrogen atoms, clean ruthenium slab, and the energy of the hydrogen molecule. The formation energy of interstitial hydrogen, normalised to the hydrogen concentration, is calculated according to the definition

(2) $$\Delta E_{H_{2}}=\left(E_{M_{x}H_{y}}-xE_{M}-\frac{y}{2}E_{H_{2}}\right)/\frac{y}{2},$$

where x,y are respectively the number of metal atoms and the number of hydrogen atoms, while $E_{M_{x}H_{y}}$, $E_{M}$, and $E_{H_{2}}$ stand respectively for the total energy of the metal hydride, the energy of each bulk metal atom, and the energy of a hydrogen molecule.

Jump frequencies for the hydrogen diffusion were extracted from the transition state calculations. The jump rate for a diffusing hydrogen atom may be expressed as

(3) $$v=v_{0}e^{-E_{b}/k_{B}T},$$

where $E_{b}$ is the energy difference between the transition state and ground state. For bulk diffusion, the pre-exponential factor $v_{0}$ in Equation (3) may be approximated by the expression [39]

(4) $$v_{0}=\frac{\prod_{i=1}^{N}\omega_{i}}{\prod_{i=1}^{N-1}\omega_{i}^{*}},$$

where $\omega_{i}$ and $\omega_{i}^{*}$ are the vibrational frequencies in the initial and transition states respectively, obtained by determining the the Hessian matrix (matrix of the second derivatives of the energy with respect to atomic positions).

Due to the low mass of the hydrogen atom, its adsorption and diffusion behaviour is, in general, influenced by zero-point energy (ZPE). The ZPE is calculated by the relation

(5) $$ZPE=\frac{\sum_{i}hv_{i}}{2},$$

where $v_{i}$ is a real normal mode frequency. The zero point energy for a hydrogen molecule (H_2_) calculated thus is 0.27 eV (0.135 eV per H atom), corresponding to a vibrational mode of 4354 cm^−1^, in good agreement with the experimentally-determined value of 4401 cm^−1^ [40]. However, ZPE contributions are not explicitly included in this work, as they do not affect the computed energies and barriers to a significant degree, particularly in relation to one another.

### 2.2. Electronic Structure and Bonding Analysis

In addition to the energy calculations, we have carried out an in-depth analysis of the chemical bonding for a thorough understanding of the interaction between species. The bonds of main interest are those between the diffusing hydrogen atom and the surface ruthenium atoms. We investigated the Bader atomic charges and volumes [41,42,43,44], the Density Derived Electrostatic and Chemical (DDEC6) bond orders and net atomic charges [45,46], the electron density and Laplacian at bond critical points (BCP) [47], and also the Crystal Orbital Hamilton Population (COHP) and Crystal Orbital Overlap Population (COOP) functions [48,49,50,51].

The Bader charge is a measure of the electron occupation on an atom, and indicates charge transfer, while the Bader volume is an indication of how closely the charge associated with each atom is localised around the nucleus. The bond-critical points (BCPs) were assigned to saddle-points of electron density along the bond-paths. The electron density value at the BCP shows the covalent energy contribution to the chemical bond. The covalent nature of the bond is reflected in the bond order, with a higher value showing a stronger covalent bond. The sum of bond orders for each atom, its total bond order, is an indication of the activity of the atom in that particular configuration. The ionic contribution of a bond can be characterized by net atomic charge, which quantifies the charge transfer between atoms. The Laplacian, the scalar derivative of the gradient vector field of the electron density, indicates where the electronic charge is locally concentrated or depleted. The sign and value of the Laplacian at the BCP provide information on the nature of a bond, with a negative sign indicating a shared interaction (e.g., covalent bonding), while a positive sign indicates a non-covalent interaction such as ionic, hydrogen, or van der Waals [52,53].

The COOP and COHP enable the extraction of information about bonding in the system on the basis of Partial Density of States (PDOS) and Bond Order Overlap Population density. They allow us to identify bonding, non-bonding, and anti-bonding interaction domains for pairs of atoms (or orbitals). The COOP is defined according to the formula:

(6) $${\mathrm{COOP}}_{ij}\left(E\right)=S_{ij}\sum_{n}c_{i}^{n}c_{j}^{*n}\delta \left(E-E_{n}\right),$$

where $S_{ij}=\langle \varphi_{i}\varphi_{j}\rangle$ is the overlap of atomic orbitals $\varphi_{i}$ and $\varphi_{j}$, and $c_{i}$ and $c_{j}$ are respectively the coefficients of these atomic orbitals in the molecular orbital $\psi_{n}$. Positive and negative COOP values indicate bonding and anti-bonding interactions, respectively, while a zero value represents a non-bonding interaction. The closely-related COHP is defined thus:

(7) $${-\mathrm{COHP}}_{ij}\left(E\right)=H_{ij}\sum_{n}c_{i}^{n}c_{j}^{*n}\delta \left(E-E_{n}\right),$$

where $H_{ij}$ represents the Hamiltonian matrix element between atomic orbitals $\varphi_{i}$ and $\varphi_{j}$. In replacing the $S_{ij}$ matrix with the $H_{ij}$, the COHP values are reversed: negative for bonding and positive for anti-bonding, with zero values again representing a non-bonding interaction. More thorough discussions of the COOP and COHP techniques can be found in the cited literature [48,49,50,51].

Combining these analytical tools allows us to carry out a comprehensive examination of the relevant bonds in each modelled system and reveal the underlining reaction mechanisms. Recently, computational studies using a combination of these electronic structure analysis techniques have led to an improved understanding of the reactivity and scaling laws on transition metal surfaces [13].

## 3. Results

In this section, we present the results of DFT calculations of hydrogen adsorption and diffusion. In the first two subsections, we report the results for hydrogen interaction with ruthenium and tin, respectively. For the former, we show the stable configurations and their energies of formation, and also present the results of CINEB transition state calculations for hydrogen diffusion and the corresponding energy barriers. In Section 3.3, to show the influence of the tin on the H–Ru interaction, we present the range of calculations performed for hydrogen–ruthenium-tin interactions on the Ru(0001) surface. The calculations cover adsorption, surface diffusion, and subsurface penetration of hydrogen.

### 3.1. Hydrogen and Ruthenium

#### 3.1.1. Surface, Subsurface, and Bulk

**Figure 1:**
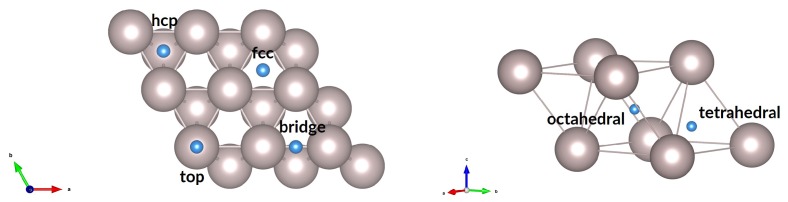
Surface adsorption and bulk interstitial sites for hydrogen on Ru(0001) and in Ru bulk. Blue spheres represent hydrogen atoms.

We calculated adsorption energies of hydrogen atoms on the ruthenium surface to identify the relative stability of different adsorption sites. The Ru(0001) surface is chosen as it has the lowest surface energy γ and is therefore the most commonly-exposed; it is well-studied and represented in the literature. We calculated the energy of adsorption of hydrogen at 14 monolayer (ML), i.e., one hydrogen atom for four surface ruthenium atoms in our (2 × 2) cell. Four sites are considered for adsorbed hydrogen atoms on the pure Ru(0001) surface, as shown in Figure 1: *top, bridge, hcp*, and *fcc* sites. We have also identified the subsurface and bulk interstitial sites for hydrogen in ruthenium. The results of the calculations are summarised in Table 1.

For Ru(0001), the *fcc* site was found to be most favourable with $E_{ads}=-0.64$ eV, a slight energy advantage of 0.06 eV relative to the *hcp* site. The top site is the least favourable at −0.15 eV. Nonetheless, the overall result for the four adsorption modes indicates that adsorption readily occurs on the ruthenium surface, as all sites have negative values for $E_{ads}$. The trend in energies and the preference for the *fcc* site is in agreement with the results reported in the literature [54,55]; the differences can be attributed to the different software, functionals, parameters, and convergence criteria used in the computations.

An interstitial hydrogen atom in ruthenium bulk can occupy one of the two sites shown in Figure 1, octahedral or tetrahedral, within the voids found in the hcp crystal structure. The results presented in Table 1 show that the interstitial sites have positive energies of formation with relative to H_2_; therefore, interstitial hydride formation is unfavourable in ruthenium at this concentration (eight ruthenium atoms per hydrogen atom). This aligns with experimental data, which show very low hydrogen solubility in ruthenium due to the highly endothermic nature of the reaction [28].

Overall, hydrogen readily adsorbs on the Ru(0001) surface, as shown by the calculated adsorption energies. According to Luppi et al., the H_2_ molecule does not face a large barrier to dissociation on the ruthenium surface: from 0.013 eV to 0.436 eV, depending on the initial configuration and exchange-correlation functional used in the computations [56]. Therefore, dissociative adsorption of molecular hydrogen on ruthenium occurs easily. However, our results show that interstitial hydrogen in the ruthenium bulk is not thermodynamically favoured.

#### 3.1.2. Diffusion

In order to acquire a more complete picture of the hydrogen–ruthenium interaction on the surface and in the bulk of the metal, we performed transition state calculations using the CINEB method. For a number of diffusion scenarios, we calculated transition states and energy barriers to hydrogen diffusion, covering (i) surface diffusion, (ii) bulk diffusion, and (iii) subsurface penetration.

For surface diffusion, we have found the transition state for diffusion of an adsorbed hydrogen atom from an *fcc* site to a neighbouring *hcp* site, and vice versa. Both transition states are found to be more or less the bridge adsorption mode, in which the hydrogen atom is equidistant from two neighbouring surface Ru atoms, above the axis joining the atoms. We found the energy barrier to be 0.18 eV for the *fcc* to *hcp* jump; the barrier for the reverse transition (*hcp* to *fcc*) is even lower, at 0.12 eV, due to the 0.06 eV difference in adsorption energies of the *hcp* and *fcc* sites. These values indicate a largely favourable energy landscape for diffusion of adsorbed hydrogen across the Ru(0001) surface, and this conclusion agrees with the findings reported elsewhere [57,58].

**Figure 2:**
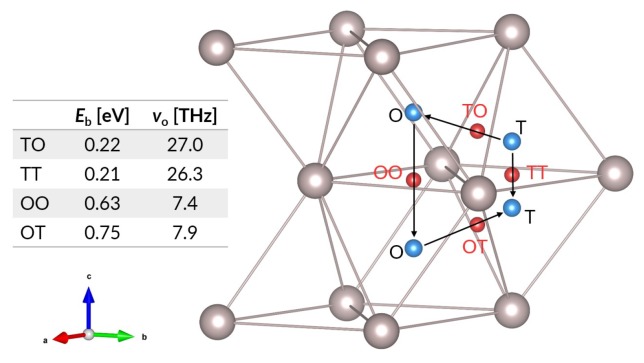
Interstitial hydrogen diffusion paths and transition states. Blue spheres represent interstitial sites, while red spheres indicate transition states. O and T correspond to octahedral and tetrahedral sites; OO, OT, TO, and TT show the corresponding diffusion path ways between these sites. The accompanying table shows energy barriers and jump frequencies (per Equation (4)) for the interstitial diffusion paths, for hydrogen concentration (H/Ru) equal to 116.

The picture for bulk diffusion is somewhat more complicated. As Figure 2 illustrates, the two stable interstitial sites (octahedral and tetrahedral sites ) imply four diffusion paths: (i) tetrahedral to octahedral (TO), (ii) tetrahedral to tetrahedral (TT), (iii) octahedral to octahedral (OO), and (iv) octahedral to tetrahedral (OT). The transition state for each path has the diffusing atom passing through a triangle formed by three ruthenium atoms. The energy barriers for all four paths are listed in Figure 2, as are the jump frequencies derived from the vibrational analyses of the interstitial hydrogen states and the transition states. The energy barriers for the jumps (0.21–0.75 eV) and the jump frequencies suggest that hydrogen diffusion in the ruthenium bulk is quite rapid. For the most important diffusion event, subsurface penetration, we have looked at one key diffusion path: from the *fcc* site on the surface to the octahedral interstitial site in the subsurface. As these sites represent the most favourable locations for hydrogen on the surface and in the subsurface void, we believe that this is the most likely path for hydrogen penetration into the metal. The energy barrier is found to be 1.06 eV, which indicates that this diffusion step is unfavourable from a thermodynamic standpoint, although it remains accessible. Most importantly, this is the largest energy barrier faced by a hydrogen atom in going from the molecule in vacuum to the metal surface, across the surface, and into the metal bulk.

### 3.2. Hydrogen and Tin

We evaluated hydrogen adsorption on tin surfaces by calculating adsorption energies for a number of sites on the Sn(100) and Sn(010) surfaces. The tin slab for both surfaces is a 1 × 1 cell of a β-Sn structure with seven layers. At 14 ML and 15 ML hydrogen coverage for the Sn(100) and Sn(010) surfaces respectively, all adsorption sites without exception have positive energies relative to H_2_, i.e., dissociation on these tin surfaces is entirely unfavourable. This points to the conclusion that tin deposited on the ruthenium surface does not provide a site of increased dissociation/adsorption of hydrogen molecules.

Tin, however, forms volatile hydrides. Stannane (SnH_4_) and possibly other hydride compounds are formed when tin is etched from a ruthenium surface by hydrogen. Stannane readily evaporates, adsorbs, and dissociates on the ruthenium surface, which results in the persistence of tin on the ruthenium surface [22,59,60].

### 3.3. The Effect of Tin

We continue our investigation of the hydrogen-tin-ruthenium interaction on the Ru(0001) surface by calculating the adsorption energies for 14 ML of tin on the Ru(0001) surface, finding that the *hcp* site is thermodynamically most favoured at this coverage, with $E_{ads}=-1.49$ eV. The adsorption energy for the *fcc* site differs by 0.09 eV from that of the *hcp* site, while the *top* site is 0.55 eV higher in energy. We found the energy barrier for tin migration from the *hcp* to the *fcc* site to be 0.13 eV. The barrier for the reverse jump is 0.04 eV; the difference is entirely due to the relative adsorption energies of the sites. Therefore, neither tin nor hydrogen faces a large energy barrier to diffusion on the Ru(0001) surface.

**Figure 3:**
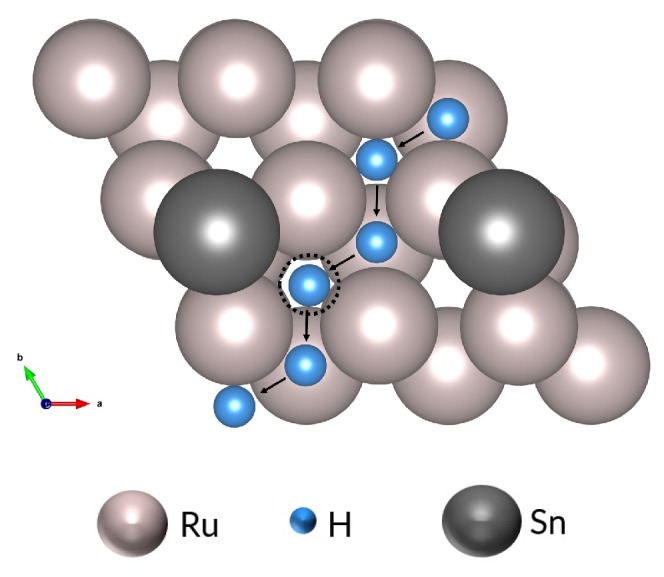
Example jump trajectory of H atom on Ru(0001) with 14 ML Sn. The highlighted site (dashed circle) is the *fcc* site next to an Sn atom (*fcc_Sn*).

We consider the adsorption and diffusion of hydrogen on the Ru(0001) surface with tin present. A tin atom is located at its preferred *hcp* site on a 2 × 2 Ru(0001) slab, and the adsorption energies are calculated for the subsequent adsorption of hydrogen to the surface. In addition to the earlier-described *top*, *hcp*, and *fcc* sites, a second type of *fcc* site (*fcc_Sn*) is found, which has the hydrogen atom in the *fcc* site closest to the Sn atom. The calculated adsorption energies are given in Table 1. As with the clean ruthenium surface, the *fcc* site is lowest in energy ($E_{ads}=-0.58$ eV); the *top* site is least favoured, with its slightly positive adsorption energy of 0.01 eV. Overall, the hydrogen atom tends to occupy sites which are not in close proximity to the tin atom. These surface adsorption results are similar to findings for PtSn surface alloys [61], on which hydrogen adsorption sites near tin are higher in energy. The *fcc_Sn* site (−0.33 eV) is higher in energy than the *hcp* and *fcc* sites, and the barrier faced by hydrogen in jumping to this site is higher than the barrier when jumping to a site not next to the tin atom. This preference for ruthenium has important consequences for hydrogen diffusion across the Ru(0001) surface in the presence of tin, as the hydrogen jump trajectory becomes more convoluted (see Figure 3) due to the change in the energy landscape and blockage of sites by tin atoms. It follows that the surface mobility of hydrogen is reduced by this obstacle, with consequences for hydrogen diffusion into the subsurface.

**Figure 4:**
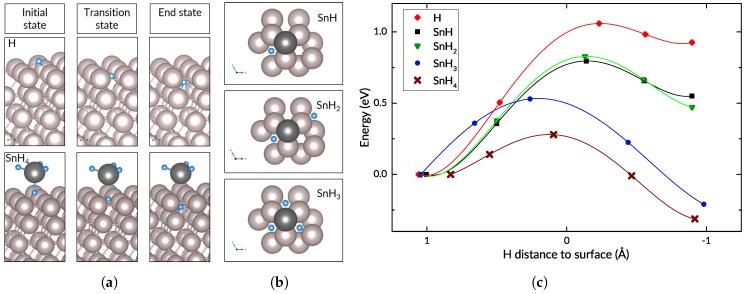
(**a**) initial, transition, and end states of hydrogen subsurface diffusion for a hydrogen atom (top row) and for SnH_4_ (bottom row) on Ru(0001); (**b**) top view of adsorbed SnH, SnH_2_, and SnH_3_ configurations; (**c**) energy profiles for diffusion of a hydrogen atom from an *fcc* site to a subsurface octahedral site. Negative distance values indicate that the H atom is beneath the ruthenium surface.

The presence of tin also results in a substantial change in the energy barrier to subsurface penetration. Figure 4 shows a comparison of the energy barriers for the migration of a hydrogen atom from the *fcc* site on the surface to a subsurface octahedral site in the presence and absence of tin. These cases correspond to adsorption and dissociation of progressively more hydrogenated tin hydrides (SnH, SnH_2_, SnH_3_, SnH_4_) or co-adsorption of tin and varying quantities of hydrogen. The energy barrier drops to 0.28 eV when the stannane molecule is the source of the diffusing atom.

It is important to note that the studied scenarios are not a simple progression from low hydrogen levels to high. The cases for SnH and SnH_2_ are very similar, differing only in the presence of a second hydrogen atom at a considerable distance from the tin atom. Their transition states are similar, with the diffusing hydrogen atom located in the centre of a triangle formed by three Ru surface atoms. The relative position of the tin atom is unchanged. However, the SnH_3_ case is different from the previous two in that its transition state and final state have the tin atom at the *fcc* site, above the penetrating hydrogen atom. In this, it is similar to the transition state of the case with the stannane molecule. Despite the clear differences in geometry and energies for all the scenarios, the initial and final positions of the diffusing hydrogen atom are the same in all cases: *fcc* site and octahedral site, respectively (see Figure 4). In all cases, the penetrating atom is found in the midst of three surface atoms in the transition state.

**Figure 5:**
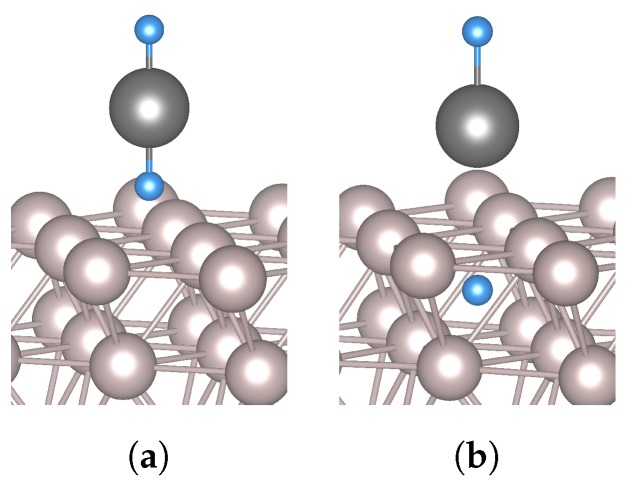
(**a**) Initial and (**b**) end states for SnH_2_* (vertically-oriented SnH_2_ molecule) on Ru.

One other modelled case, which differs from all of those described in the preceding paragraph, is the case for a vertically-oriented SnH_2_ molecule (SnH_2_*), with a hydrogen atom above the *fcc* site. As shown in Figure 5, the molecule does not adsorb on the Ru(0001) surface; rather, at the end of the relaxation calculation, the hydrogen atom which is initially closer to the metal surface is located in the octahedral subsurface site. The tin atom ends up at the *fcc* site, with the second hydrogen atom adsorbed on top of the tin atom. Therefore, what our calculations show is a more or less barrier-less penetration through the surface when the molecule approaches the surface in this configuration.

As mentioned earlier, the energy barrier to subsurface penetration for hydrogen on an otherwise clean Ru(0001) surface equals 1.06 eV. When tin is present, the energy barrier is lower in all the different test cases: 0.80 eV for SnH, 0.83 eV for SnH_2_, 0.53 eV for SnH_3_, and 0.28 eV when the hydrogen atom is taken from the adsorbed SnH_4_ molecule, which is a considerable drop from the value for hydrogen on Ru(0001) with no tin present. For subsurface penetration at sites far from the tin atom, the energy barrier is essentially unchanged. In other words, the diffusion of a hydrogen atom from the *fcc* site on the Ru(0001) surface to the underlying octahedral void in the subsurface is made significantly easier by the proximity of a tin atom.

### 3.4. Charge Density Analysis

**Figure 6:**
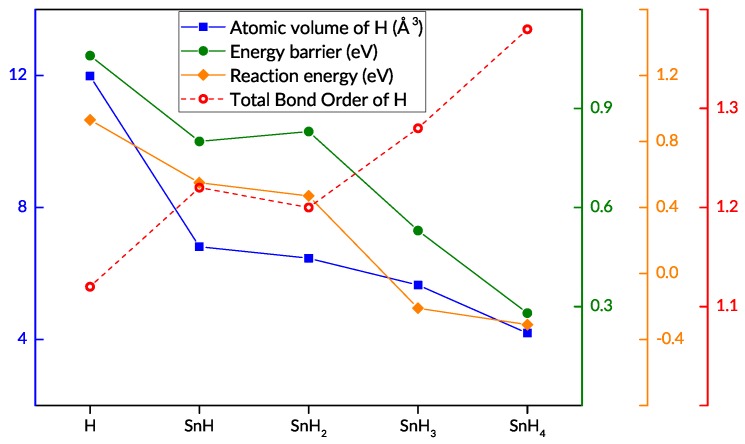
Energy barrier, formation energy, atomic volume and sum of the bond orders of the diffusing hydrogen atom in the initial state for each modelled diffusion scenario.

A deeper look into the effect of tin on the subsurface penetration is obtained by analysing the electronic charge density, based on the output of the DFT calculations. In the various penetration scenarios, the bonds which are formed and broken affect the energy barrier to H penetration, and so do the electronic configurations of the participating H, Sn, and Ru surface atoms. Via bond order, Bader charge, and topological analyses, we examined the changes in charge density which accompany the changes in the energy barriers. The main focus is on the bonds between the diffusing hydrogen atom and the three surrounding ruthenium atoms, because of their direct relevance to the transition from the surface *fcc* site to the subsurface site, and the analyses reveal trends in various characteristics of the Ru–H bonds in the initial adsorbed state (Table 2). The salient ones—energy barrier, reaction energy, atomic volume and bond order of the hydrogen atom—are plotted in Figure 6 for each diffusion scenario we explored. We find that the sum of bond orders for hydrogen is inversely proportional to the barrier to diffusion, while a smaller H atomic volume and more negative reaction energy correspond to lower diffusion barriers. We consider these trends and their implications in more detail in the Discussion section.

**Figure 7:**
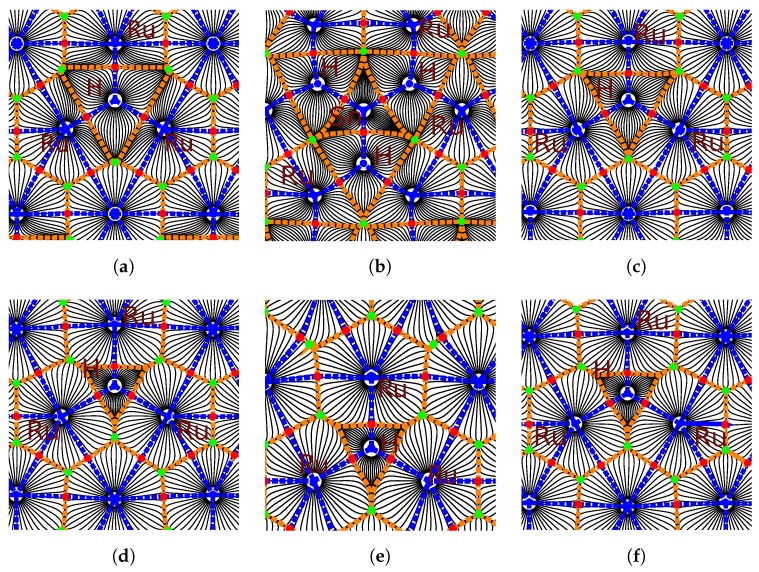
Topological analysis of the electron density for (**a**–**c**) H on Ru(0001), SnH_3_ on Ru, and SnH_4_ on Ru, respectively; (**d**–**f**) transition states with H from the adsorbate (H, SnH_3_, and SnH_4_ respectively) between three ruthenium surface atoms. The figures show the horizontal plane through the diffusing hydrogen atom. Atomic basins are circumscribed by dashed orange lines. Within each atomic basin, the blue dot marks the position of the nucleus. Bond paths are shown with dashed blue lines. Along each bond path, the red dot represents the bond critical point (BCP). The green dots represent the ring critical points.

An illustration of one of the differences in bonding is provided in Figure 7. The topological analysis of the electron density distribution shows a symmetrical allocation of bonds between the H atom in the *fcc* position and three surrounding Ru atoms on the Ru(0001) surface (Figure 7a). The central triangle shows the extent of the electron density associated with the adsorbed H atom. When an SnH_4_ molecule is adsorbed on the surface, with one hydrogen atom oriented downwards, the bonds formed between the ruthenium atoms and the hydrogen atom are nearly identical to those formed by the lone H atom, with the important difference being the bond critical points’ location closer to the hydrogen atom; the triangle demarcating the H atom’s basin is perceptibly smaller in area in Figure 7c (SnH_4_ on Ru) than in Figure 7a (H on Ru). This indicates that the charge associated with the hydrogen atom is located in a smaller region, and this is confirmed by the calculated atomic volume of hydrogen (Table 2, Figure 6). In the transition state, in which the hydrogen atom is surrounded by three (3) Ru atoms in the plane of the Ru(0001) surface, the electron density concentrated around the diffusing hydrogen atom is confined within similar size volumes, irrespective of the structure considered (Figure 7d–f).

**Figure 8:**
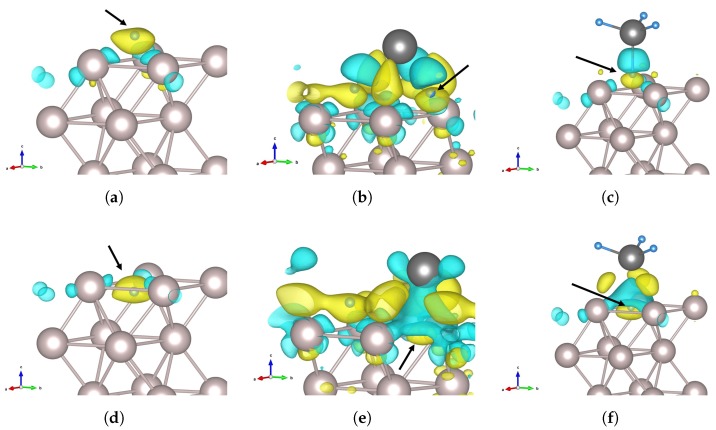
Charge difference for (**a**–**c**) H on Ru(0001), SnH_3_ on Ru, and SnH_4_ on Ru, respectively; (**d**–**f**) transition states with H from the adsorbate (H, SnH_3_, and SnH_4_, respectively) between three ruthenium surface atoms. In all the figures, the yellow isosurface is for charge density −0.004 e Å^−3^, and shows regions of charge accumulation, while the cyan is for +0.004 e Å^−3^, and shows regions of charge depletion. The black arrow shows the location of the diffusing hydrogen atom.

Charge density difference (Δρ) plots paint a three-dimensional picture of the influence of tin on the charge density distribution. Figure 8 shows the charge difference for the initial and transition states for the H, SnH_3_, and SnH_4_ cases. In Figure 8a, which shows an adsorbed H atom at the *fcc* site on the Ru(0001) surface, the H atom is surrounded by a region of accumulation; this indicates a net transfer of charge density to the hydrogen from the areas of depletion around the three surrounding Ru atoms. There are also small regions of increased density near the Ru atoms, beneath the surface. The transition state in Figure 8d is quite similar, with smaller regions of depletion on the Ru atoms. For the SnH_4_ case (Figure 8c,d), the charge density donated to the hydrogen’s 1*s* orbital is taken not only from the ruthenium, but in large part from the region between the H and Sn atoms. This points to the transfer of charge to the more electronegative hydrogen. Furthermore, the small regions of charge accumulation on the Ru atoms are now positioned above the ruthenium surface. The transition state shows redistribution of the charge to regions between the Ru atoms and the Sn atom, with a large depletion region above the penetrating H atom.

Due to the number of atoms on the ruthenium surface, SnH_3_ on Ru presents the most complex interactions, which is evident from Figure 8b,e. The donation to the hydrogen is again present, but the source of the transferred charge is different. Each ruthenium atom on the surface has several small depletion zones, but the charge redistribution has resulted in large accumulation zones between the Sn atom and the surrounding Ru atoms, interleaved with depletion zones above the adsorbed hydrogen atoms, which are also surrounded by areas of increased charge density. There appears to be significant charge transfer from the Sn atom, both to the hydrogen atoms and the surface ruthenium atoms. In the transition state, a large depletion zone similar to that found in Figure 8f is found above the diffusing hydrogen atom, also surrounded by zones of increased charge density. The implication is that electron–electron repulsion in the SnH_3_ and SnH_4_ cases makes the transition state more accessible, and makes the end state more energetically favourable, which is reflected in the exothermic nature of the reactions.

**Figure 9:**
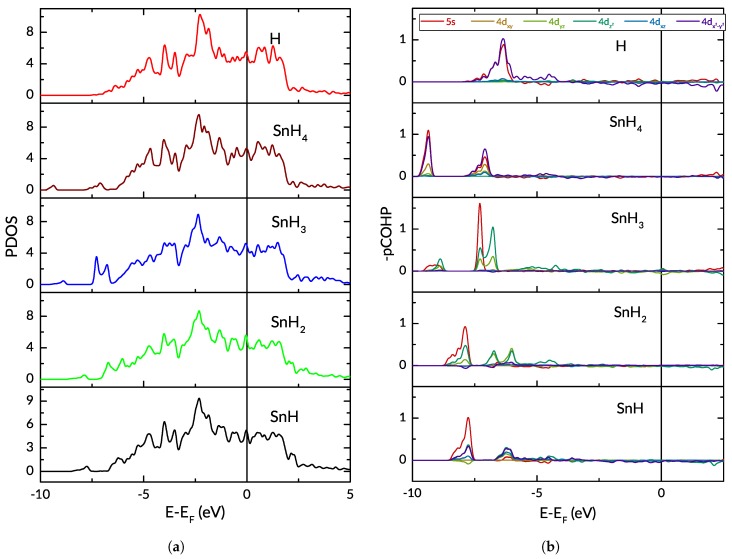
(**a**) projected Density of States for *d*-band of ruthenium surface atoms with different adsorbates; and (**b**) projected COHP for Ru–H interaction. i.e., one ruthenium atom and the adsorbed hydrogen atom. For clarity, the COHP for the weakest interactions are not shown. In all cases, the initial structure is considered.

Using the LOBSTER code [51], we performed density-of-states and COHP/COOP calculations. We extracted the total and projected densities of states for the various cases and computed the orbitalwise COHP and COOP for the hydrogen atom and the surface ruthenium atoms. In Figure 9, we present the projected densities of states (PDOS) curves for the *d*-band electrons of the ruthenium atoms on the surface, with different adsorbates; the COHP for the interaction between the key hydrogen atom and one of the three surrounding ruthenium atoms are also given.

The main differences in the density-of-states can be observed in the lowest energy levels (below −5 eV), where the adsorbed hydrogen and tin atoms cause the appearance and/or shift of peaks. The centre of the *d* band distribution is lower when the Sn atom is adsorbed on the Ru(0001) surface, and moves progressively lower with the adsorption of more hydrogen atoms. The *d* band width increases as the band centre is shifted downward. In the COHP plots (–pCOHP, to be precise), significant differences can be seen in the orbitals which contribute to the bonding between the atoms. We show in Figure 9 the COHP for the key hydrogen atom and one of the surface Ru atoms surrounding it. Whereas both the 5s and $4d_{x^{2}-y^{2}}$ orbitals dominate the bonding and very slight anti-bonding populations for the H and SnH_4_ adsorbates, the 5s contribution is much reduced for the other cases with a tin atom on the ruthenium surface, in which the $4d_{z^{2}}$ interaction with the hydrogen comes to prominence as more hydrogen atoms are added. The COHP for the other Ru–H pairs in the same scenario are not necessarily identical to those in Figure 9 because the tin affects the surrounding Ru atoms to different degrees, based on their distance and arrangement. Taken together, the COHP and the Δρ plots of Figure 8 confirm that the electrons participating in the bonding generally originate from different orbitals for different adsorbates. This is especially apparent when the SnH_3_ case is compared to the others.

## 4. Discussion

A number of observations can be made upon comparing the results of the charge density analyses with the results of the NEB calculations (Table 2). First, it can be seen that the energy barrier is higher when the distance between the adsorbed H atom and each of the surface Ru atoms is greater; topological analyses show that the same is true for the distance between the H atom and the bond critical point. The difference is not stark: the adsorbed SnH_4_ has the hydrogen atom in the *fcc* site at an average distance 4% smaller than the average distance between a lone H atom and the surrounding Ru atoms; the difference in the H-BCP distance is 9%. The barrier–distance relationship is not necessarily enough to predict a difference in energy barriers, since the three cases with Sn coadsorbed with H on the surface (SnH, SnH_2_, and SnH_3_ in Table 2) have the same bond lengths and BCP distances, but different barriers to penetration.

A second, clearer correlation can be observed between the total bond order of the hydrogen atom and the calculated energy barrier. Taking into account all of the bonds in which the hydrogen atom of interest participates, we have calculated the sum of bond orders, and find that the changes in this value correspond to the changes in energy barrier quite neatly: a larger total bond order for H corresponds to a lower subsurface penetration barrier. The total bond order of the hydrogen atom in the SnH and SnH_2_ cases are quite close, 1.22 and 1.20, respectively, as are the energy barriers, 0.80 and 0.83 eV, respectively. Moreover, the inverse proportionality is maintained. The trend holds for all the examined scenarios, suggesting that a key factor in the observed barrier differences is the state of the adsorbed and subsequently diffusing hydrogen atom itself, as characterised by its bonds and associated electron density.

The results of Bader charge analysis on the charge density shows a third trend, which relates the charge density distribution and the energy barrier. Recall that Bader’s definition of an atom uses zero-flux surfaces to divide atoms, thereby associating a certain region of the electronic charge density with each atom in the system. In our system, the atomic volume computed for the adsorbed H atom of interest is generally larger for the cases with higher energy barriers, with the smallest volume corresponding to the lowest energy barrier. This supports the inference that the smaller atomic volume of H in a certain adsorption mode leads to a lower energy barrier to subsequent diffusion into the subsurface. We can discern yet another link when comparing the energy barriers to the reaction energies, i.e., the total energy difference between the initial adsorbed state and the final state with hydrogen in the subsurface. Whereas the subsurface penetration event is endothermic for a hydrogen atom on Ru(0001), it is made progressively less so in the presence of tin, and is ultimately exothermic when the hydrogen is taken from a stannane molecule. Moreover, as seen in Figure 4, the SnH_3_ and SnH_4_ cases have early transition states, which contrasts with the late transition states of the other cases.

We note that some of the other characteristics we examined do not show such a clear trend when taken in isolation. For instance, the charge on the H atom as calculated by the Bader method does not match the trend in energy barriers. The trend of net atomic charges (NAC) calculated via the DDEC6 approach is also inconclusive, as are the trends of electronic density (ED) and charge density Laplacians at the bond critical point. Nevertheless, viewed together with the trends found in the hydrogen BCP, bond order, and atomic volume, these characteristics indicate that the hydrogen atom is in a state with more localised charge in the scenarios with lower penetration barriers. In the transition states, the area of the triangle formed by the surrounding ruthenium atoms is in fact smaller for the SnH_4_ case than for the lone hydrogen atom. It appears that the localisation of the charge around the H nucleus is of more importance than the actual charge on the atom.

We speculate that the lower electronegativity of tin (1.96) relative to hydrogen (2.2) leads to the more compact localisation of charge around the hydrogen nucleus, i.e., the shared electrons are drawn closer to the hydrogen atom, which results in the reduced atomic volume found in our calculations. The introduction of tin results in charge transfer to the hydrogen atom, which can be seen in the more negative values of net atomic charge (NAC in Table 2) of the hydrogen atom in the scenarios with tin. Ruthenium’s electronegativity is 2.2, equal to that of hydrogen, and the ruthenium atoms also show a more negative net atomic charge when tin is present. The tin atom therefore donates charge to the hydrogen and ruthenium atoms, as made evident by its positive NAC, and the charge accumulation above the ruthenium surface pushes the hydrogen into the surface due to electron–electron repulsion. The charge accumulation and repulsion also account for the preference of hydrogen for adsorption sites farther from tin, as seen in Section 3.3. The lower electronegativity of tin has also been put forward as the key factor in the difficulty of tin removal from ruthenium using hydrogen [60].

The barrier-less penetration of the Ru(0001) surface by the hydrogen atom from the vertically-oriented SnH_2_ appears to support the conclusion that the hydrogen atom is in a decisively different state of bonding and charge localisation when the subsurface penetration is more facile. Both the total bond order and Bader volume (see Table 2) suggest that the energy barrier would be lower in this case than in the SnH_4_ scenario; the bond order is 1.54, the highest value, while the Bader volume is 3.54, the lowest value. The charge difference (Δρ) in the initial geometry of the relaxation also shows charge accumulation around the hydrogen and above the ruthenium surface, in a manner similar to the transition state for SnH_4_. Indeed, this calculation finds its local minimum with the hydrogen atom below the surface, showing that the hydrogen atom attached to tin is in a most advantageous state for passing through the surface ruthenium atoms.

These results together indicate that a closer, shorter bond exists between the hydrogen atom and the surface ruthenium atoms in the presence of tin, with a smaller atomic volume for the H atom in the adsorbed state, which is corroborated by the trend in calculated Bader volumes. The surrounding ruthenium atoms are moved apart to a lesser extent (smaller change in area of the triangle, see Table 2) to arrive at the transition state, i.e., at a lower energy cost. The reduced atomic volume is likely due to the asymmetric charge distribution relative to the less electronegative tin. The charge transfer from tin to hydrogen and ruthenium creates areas of charge accumulation around the hydrogen atom and on the ruthenium surface, and makes the subsurface interstitial site more energetically favourable. These conditions are most advantageous for the adsorbed SnH_3_ and SnH_4_, with the result that the penetration of hydrogen is easiest in these cases.

## 5. Conclusions

In this work, we have investigated hydrogen penetration of the Ru(0001) surface in the presence and absence of tin, by means of DFT computations, chemical bonding, and charge density analysis. We showed that hydrogen faces a significant barrier to subsurface penetration in the absence of tin. We find that the energy barrier drops significantly in the presence of tin, be it as an adatom or as part of an adsorbed tin hydride molecule. The lowest energy barriers are found when a hydride molecule adsorbs on the Ru(0001) surface and subsequently loses a hydrogen atom. We performed charge density analyses on systems with and without tin, which revealed changes in the bonds formed between ruthenium and hydrogen under the influence of tin, as well as a change in charge distribution around the diffusing hydrogen atom, resulting in reduced energy barriers to penetration. The much higher barrier found for a hydrogen atom with no tin present allows us to conclude that tin hydrides play an important role in the increased blistering of EUV reflectors with tin debris on the surface.

These insights into the effect of charge transfer from tin have important implications for the development of blistering mitigation techniques, and prolonging the operational lifetime of EUV optics. Our findings may also have value in cases where hydrogen penetration needs to be improved, such as hydrogen storage or separation and purification.

## Figures and Tables

**Table 1:** Energies and structure parameters for adsorbed and interstitial hydrogen on Ru(0001) and in ruthenium bulk. Adsorption energies correspond to 14 ML hydrogen coverage, while hydride formation energies are for hydrogen concentration (H/Ru) equal to 18. Sites marked “*” correspond to calculations done with one (1) Sn adatom in an *hcp* site; *fcc_Sn* is the *fcc* site closest to the Sn adatom in an hcp site.

	$E_{\mathrm{ads}}$ [eV]				$\Delta E_{H_{2}}$ [eV]	
	This Work	Other			This Work	Other
*top*	−0.15	−0.14 [55], −0.09 [54]		T_bulk_	0.76	
*bridge*	−0.45	−0.44 [55], −0.43 [54]		O_bulk_	0.21	
*hcp*	−0.58	−0.50 [55], −0.52 [54]		T_sub_	0.90	1.04 [55]
*fcc*	−0.64	−0.59 [55], −0.55 [54]		O_sub_	0.15	0.37 [55]
*top* *	0.01					
*hcp* *	−0.52					
*fcc* *	−0.58					
*fcc_Sn* *	−0.33					

**Table 2:** Characteristics of diffusing H atom and bonds between H atom and surrounding Ru surface atoms in the initial state. Values for SnH_2_* do not correspond to clearly-defined initial and transition states (see text). Inter-atomic distances ($d_{H-Ru}$), bond critical point (BCP) distances ($d_{H-BCP}$), electron density and its Laplacian at BCPs are average values for 3 Ru–H bonds. Net atomic charge (NAC) for Ru is sum over 4 surface Ru atoms in (2×2) unit cell.

	$d_{H-\mathrm{Ru}}$/ $d_{H-\mathrm{BCP}}$ [Å]	Total Bond Order	Electron Density	Laplacian	Bader Volume [Å^3^]	NAC			Reaction Energy [eV]	Barrier [eV]
						H	Ru	Sn		
H	1.90/0.69	1.12	0.50	2.79	11.98	−0.051	−0.01	-	0.93	1.06
SnH	1.89/0.68	1.22	0.50	3.59	6.81	−0.059	−0.26	0.22	0.55	0.80
SnH_2_	1.88/0.68	1.20	0.52	3.56	6.46	−0.061	−0.20	0.25	0.47	0.83
SnH_3_	1.89/0.68	1.28	0.51	3.53	5.65	−0.075	−0.14	0.33	−0.21	0.53
SnH_4_	1.82/0.63	1.38	0.56	3.86	4.19	−0.062	−0.23	0.57	−0.31	0.28
SnH_2_ *	1.93/0.70	1.54	0.45	3.54	3.54	−0.061	−0.20	0.25	-	-
